# CD19/CD22 dual‐targeting chimeric antigen receptor T‐cell therapy bridging to allogeneic haematopoietic stem cell transplantation for B‐cell acute lymphoblastic leukaemia delays platelet recovery and increases risks of cytomegalovirus and Epstein–Barr virus viremia after transplantation

**DOI:** 10.1002/ctm2.1459

**Published:** 2023-10-26

**Authors:** Shijia Li, Jianrong Ge, Shiyuan Zhou, Wenjuan Zhu, Yue Han, Suning Chen, Shengli Xue, Ying Wang, Huiying Qiu, Xiaojin Wu, Depei Wu

**Affiliations:** ^1^ National Clinical Research Center for Hematologic Diseases Jiangsu Institute of Hematology, The First Affiliated Hospital of Soochow University Suzhou China; ^2^ Collaborative Innovation Center of Hematology Institute of Blood and Marrow Transplantation, Soochow University Suzhou China

Dear Editor,

Chimeric antigen receptor T (CAR‐T) cell therapy has been one of the most promising strategies to achieve improved complete remission (CR) in patients with refractory/relapsed B‐cell acute lymphoblastic leukaemia (B‐ALL) but remissions can be short‐lived.^1^ Increasing evidence gathered from recent studies has shown that CAR‐T‐cell therapy alongside complementary allo‐haematopoietic stem cell transplantation (HSCT) may achieve more durable long‐term remission than CAR‐T‐cell therapy alone.^2^ However, whether this combinational therapy is capable of exerting its impact on HSCT‐related complications remains unclear.

Therefore, we retrospectively investigated 169 consecutive patients who received allo‐HSCT for B‐ALL after CR between June 2017 and February 2022 in our department. Twenty‐nine patients received CD19 and CD22 dual‐targeting CAR‐T therapy prior to HSCT (CD19/CD22 dual‐targeting CAR‐T group), 43 patients received CD19 single‐targeting CAR‐T‐cell therapy prior to HSCT (CD19 single‐targeting CAR‐T group) and 97 patients did not receive CAR‐T therapy prior to transplantation (non‐CAR‐T group). CAR‐T generation and infusion, conditioning regimens, graft versus host disease (GVHD) prophylaxis and statistical methods are described in the Supporting Information.

The detailed characteristics of the three groups and transplant outcomes are summarised in Tables S1 and S2.

There were no differences in cumulative incidences of neutrophil engraftment among the three groups (*p* = .63) (Figure 1A). The median time to neutrophil engraftment did not differ among the three groups (*p* = .80; Table S2). After 28 days, the cumulative incidence of platelet engraftment was 69.0% (95% confidence interval [CI]: 46.6%–82.0%) in the CD19/CD22 dual‐targeting CAR‐T group compared to 87.4% (95% CI: 78.6%–92.6%, *p* = .008) in the non‐CAR‐T group and 83.7% (95% CI: 67.9%–91.7%, *p* = .053) in the CD19 single‐targeting CAR‐T group (Figure 1B). The median delay of platelet engraftment was significantly longer in the CD19/CD22 dual‐targeting CAR‐T group than in the non‐CAR‐T group (*p* = .007) and the CD19 single‐targeting CAR‐T group (*p* = .076) (Table S2). Univariate and multivariate analyses of failure to achieve platelet engraftment were performed and demonstrated that CD19/CD22 dual‐targeting CAR‐T therapy was an independent risk factor for slower 28‐day platelet recovery (odds ratio: 2.80, 95% CI: 1.03–7.30, *p* = .030) (Table 1).

**FIGURE 1:**
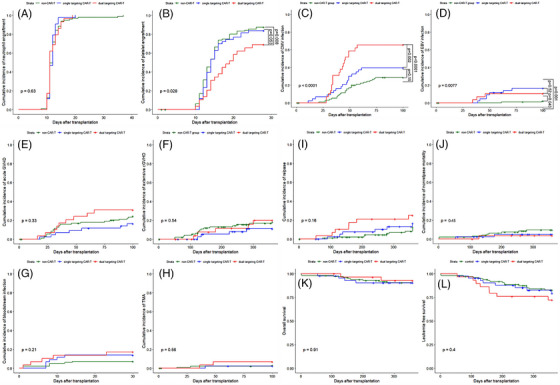
(A) Cumulative incidence of neutrophil engraftment among the three groups (*p* = .63). (B) Cumulative incidence of platelet engraftment among the three groups (*p* = .028). (C) Cumulative incidence of cytomegalovirus (CMV) infection among the three groups (*p* < .0001). (D) Cumulative incidence of Epstein–Barr virus (EBV) infection among the three groups (*p* = .0077). (E) Cumulative incidence of acute graft versus host disease (aGVHD) among the three groups (*p* = .33). (F) Cumulative incidence of chronic graft versus host disease (cGVHD) among the three groups (*p* = .54). (G) Cumulative incidence of bloodstream infection among the three groups (*p* = .21). (H) Cumulative incidence of thrombotic microangiopathy (TMA) among the three groups (*p* = .56). (I) One‐year cumulative incidence of relapse after transplantation among three groups (*p* = .16). (J) One‐year cumulative incidence of non‐relapse mortality after transplantation among the three groups (*p* = .45). (K) One‐year overall survival after transplantation between the three groups (*p* = .91). (L) One‐year leukaemia‐free survival after transplantation among the three groups (*p* = .40).

**TABLE 1:** Univariate and multivariate logistic regression analyses for failure to achieve platelet engraftment at 28 days after transplantation.

	Univariate analysis			Multivariate analysis		
Characteristics	Odds ratio	95% CI	*p*‐Value	Odds ratio	95% CI	*p*‐Value
Dual‐targeting CAR‐T versus others	2.55	1.02–6.36	**.045**	2.80	1.03–7.30	**.030**
Gender (male vs. female)	1.56	.69–3.52	.283			
Age	1.02	.99–1.06	.155			
Disease status (CR ≥2 vs. CR 1)	1.26	.57–2.78	.569			
CD34 count (≥median vs. <median)	.44	.19–1.00	**.049**	.38	.16–.90	**.027**
MNC count (≥median vs. <median)	1.2	.44–3.25	.723			
Donor–recipient gender match (female to male vs. others)	.45	.10–2.05	.303			
Type of donor (Haplo‐d vs. others)	3.15	.90–11.00	.073			
ABO compatibility (major or bidirectional incompatibility vs. others)	1.67	.71–3.93	.239			
aGVHD	21.23	2.28–197.65	.007	27.11	3.59–559.39	.005

Abbreviations: ABO, ABO blood type; aGVHD, acute graft versus host disease; CAR‐T, chimeric antigen receptor T; CI, confidence interval; CR, complete remission; MNC, Mononuclear cells.

The 100‐day cumulative incidence of cytomegalovirus (CMV) viremia was 65.5% (95% CI: 43.1%–79.1%) in the CD19/CD22 dual‐targeting CAR‐T group compared to 28.6% (95% CI: 18.9%–37.2%, *p* < .001) in the non‐CAR‐T group and 39.5% (95% CI: 23.0%–52.5%, *p* = .002) in the CD19 single‐targeting CAR‐T group (Figure 1C). The occurrence of CMV viremia in the first 100 days after transplantation did not differ significantly between the CD19 single‐targeting CAR‐T‐cell group and non‐CAR‐T‐cell group (*p* = .52). According to univariate and subsequent Cox multivariate analyses, the CD19/CD22 dual‐targeting CAR‐T therapy prior to HSCT remained significantly associated with CMV viremia in the first 100 days after HSCT (hazard ratio [HR]: 2.96, 95% CI: 1.46–4.81, *p* = .001) (Table 2).

**TABLE 2:** Univariate and multivariate Cox regression analyses for cytomegalovirus (CMV) infection during the first 100 days after haematopoietic stem cell transplantation.

	Univariate analysis			Multivariate analysis		
Characteristics	Hazard ratio	95% CI	*p*‐Value	Hazard ratio	95% CI	*p*‐Value
Dual‐targeting CAR‐T versus control	2.96	1.72–5.09	.0001	2.65	1.46–4.81	**.001**
Gender (male vs. female)	.93	.57–1.52	.768			
Age	.99	.97–1.01	.496			
Disease status (CR ≥2 vs. CR 1)	1.49	.91–2.44	.114			
CD34 count (≥median vs. <median)	1.38	.76–2.50	.290			
MNC count (≥median vs. <median)	1.03	.54–1.97	.935			
Donor–recipient gender match (female to male vs. others)	.93	.42–2.04	.859			
Type of donor (Haplo‐d vs. others)	1.86	.92–3.76	**.086**	1.66	.81–3.38	.163
ABO compatibility (major or bidirectional incompatibility vs. others)	.70	.37–1.32	.270			
aGVHD before CMV infection	1.44	.81–2.53	.213			
Cytokine release syndrome (grade 2–3)	1.83	.98–3.44	**.059**	1.19	.59–2.37	.622

Abbreviations: ABO, ABO blood type; aGVHD, acute graft versus host disease; CAR‐T, chimeric antigen receptor T; CI, confidence interval; CR, complete remission; MNC, Mononuclear cells.

The 100‐day cumulative incidence of Epstein–Barr virus (EBV) viremia was 10.3% (95% CI: 0%–20.8%) in the CD19/CD22 dual‐targeting CAR‐T group compared to 2.1% (95% CI: 0%–5.0%, *p* = .043) in the non‐CAR‐T group (Figure 1D). The 100‐day cumulative incidence of EBV viremia was 18.9% (95% CI: 6.2–29.8) in the CD19 single‐targeting CAR‐T group compared to 2.1% (95% CI: 0%–5.0%, *p* = .001) in the non‐CAR‐T group (Figure 1C). There was no significant difference in EBV infection between the CD19 single‐targeting and the CD19/CD22 dual‐targeting CAR‐T groups (*p* = .37; Figure 1D). Patients who received CAR‐T‐cell transfusion ahead of HSCT had increased 100‐day cumulative incidence of EBV viremia compared with patients in the non‐CAR‐T group (15.4% vs. 2.1%, *p* = .001). Upon univariate and multivariate analyses, the application of CAR‐T therapy was demonstrated to be the only independent risk factor for the 100‐day EBV viremia after transplantation (HR: 8.52, 95% CI: 1.48–49.1, *p* = .017) (Table 3).

**TABLE 3:** Univariate and multivariate Cox regression analyses for Epstein–Barr virus (EBV) infection during the first 100 days after haematopoietic stem cell transplantation.

	Univariate analysis			Multivariate analysis		
Characteristics	Hazard ratio	95% CI	*p*‐Value	Hazard ratio	95% CI	*p*‐Value
CAR‐T vs. control	7.95	1.76–35.89	**.007**	8.52	1.48–49.15	**.017**
Age	1.01	.97–1.06	.563			
Gender (male vs. female)	1.95	.6–6.32	.268			
CD34 count (≥median vs. <median)	.44	.15–1.32	.145			
MNC count (≥median vs. <median)	.86	.19–3.88	.844			
Donor–recipient gender match (female to male vs. others)	2.12	.58–7.69	.255			
Type of donor (Haplo‐d vs. others)	3.73	.49–28.7	.206			
ABO compatibility (major or bidirectional incompatibility vs. others)	.89	.24–3.23	.856			
Disease status (CR ≥2 vs. CR 1)	3.27	1.01–10.64	**.048**	.87	.21–3.50	.847
Cytokine release syndrome (grade 2–3)	3.23	.99–10.49	.051			
aGVHD before EBV infection	3.47	1.12–10.75	**.031**	3.99	1.27–12.55	.018

Abbreviations: ABO, ABO blood type; aGVHD, acute graft versus host disease; CAR‐T, chimeric antigen receptor T; CI, confidence interval; CR, complete remission; MNC, Mononuclear cells.

The incidence of GVHD, bloodstream infection, thrombotic microangiopathy and prognosis are described in the Supporting Information.

Our results indicated that CD19/CD22 dual‐targeting CAR‐T therapy slowed platelet recovery. Previous studies have shown that platelet engraftment was significantly delayed in an anti‐CD19 single‐targeting CAR‐T‐allo‐HSCT group compared to a non‐CAR‐T‐allo‐HSCT group.^3^ Bone marrow microenvironment abnormalities, immune microenvironment abnormalities and impaired expression of bone marrow endothelial cells as well as related chemokines that are responsible for the migration and differentiation of megakaryocytes can lead to the occurrence of thrombocytopenia after transplantation.^4^ Here, CAR‐T‐cell transfusion might cause a perturbation of immune reconstitution and thereby modulate the immune microenvironment, leading to endothelial cell damage with the potential to interfere with the mechanisms mentioned above, resulting in disturbed platelet reconstitution.^5^, ^6^

Infections remain a common complication not only in CAR‐T therapy but also in HSCT. Our study demonstrated the adverse effects of CAR‐T therapy before HSCT on CMV and EBV reactivation by day 100 after transplantation. Viral infections have been a common cause of infection in febrile CAR‐T recipients.^7^ Other studies showed hypogammaglobulinemia was found in 35% of adult patients between days 15‐30, 27% between days 31‐60, and 67% at 90 days or later after CAR T‐cell infusion, respectively.^8^ The median time interval from the CAR‐T therapy to HSCT in our study was approximately 2 months and the impact of CAR‐T therapy leading to hypogammaglobulinaemia could be pre‐transplant or post‐transplant. Researches also showed that pre‐transplant immunoglobulin G under 800 mg is an independent risk factor for hypogammaglobulinaemia after allo‐stem cell transplantation (HR: 1.81, 95% CI: 1.02–3.33, *p* = .04).^9^ Therefore, integrating CAR‐T and HSCT may result in severe post‐transplant hypogammaglobulinaemia for risks of CMV and EBV infections. A low CD4 count after CAR‐T therapy has also been documented for up to 1‐year post‐infusion, which is correlated with significantly longer duration of B‐cell aplasia.^10^ Without full recovery of the immune system, most patients received sequential HSCT with a myeloablative conditioning regimen and immunosuppressive therapy for GVHD, which further aggravated humoral and cellular immune impairment caused by CAR‐T therapy and posed patients with a high risk for viral infection.

The present study strongly indicates that integration of dual‐targeting CAR‐T therapy and HSCT contributes significantly to delaying platelet engraftment, as well as early CMV and EBV viremia. The significance and limitations of this study are discussed in detail in the Supporting Information.

## CONFLICT OF INTEREST STATEMENT

The authors declare they have no conflicts of interest.

## Supporting information

Supporting Information

## Data Availability

The original contributions presented in the study are included in the article/Supporting Information. Further inquiries can be directed to the corresponding authors.
