# Supplementary material for: CD19/CD22 dual‐targeting chimeric antigen receptor T‐cell therapy bridging to allogeneic haematopoietic stem cell transplantation for B‐cell acute lymphoblastic leukaemia delays platelet recovery and increases risks of cytomegalovirus and Epstein–Barr virus viremia after transplantation
Source: Clin Transl Med. 2023 Oct 26;13(10):e1459. doi: 10.1002/ctm2.1459 (PMC10600828; doi:10.1002/ctm2.1459)
Supplement: Supplementary file 1 — Supporting Information [file CTM2-13-e1459-s001.docx]

Supplementary Methods

Patients

Patients were eligible if they achieved CR before HSCT. All patients provided written informed consent prior to the start of this study in accordance with the Declaration of Helsinki and the approval from the Faculty Hospital Ethics Committee at the First Affiliated Hospital of Soochow University.

Humanized CAR-T generation and infusion

To create the dual-targeting CD19 and CD22 CAR-T cells and the single-targeting CD19 CAR-T cells, autologous lymphocytes were isolated from patients. T cells were sorted and expanded using CD3 magnetic beads and transduced using recombinant lentiviral viruses encoding the the CD19/22-targeted CAR and CD19-targeted CAR. Cells were then cultured in X-Vivo 15 medium containing interleukin (IL)-7 and IL-15 cytokines (The Unicar-Therapy Bio-medicine Technology Co., Ltd, Shanghai, China). The lymphodepletion conditioning regimen involved fludarabine at a level of 30 mg/m2/day and cyclophosphamide at a level of 300 mg/m2/day both from days −4 to −2 before a split-dose infusion of humanized CD19-targeted CAR-T on days 1 and 2 (40% of the total dose on day 1 and 60% on day 2). 2 patients in the dual-targeting CAR-T group and 27 patients in the single-targeting CAR-T group received an overall dose of 5*106/kg. 26 patients in the dual-targeting CAR-T group and 16 patients in the single targeting CAR-T group received an overall dose of 10*106/kg. 1 patient in the dual-targeting CAR-T group received an overall dose of 20*106/kg.

Clinical transplant protocol

Most patients were pretreated with a modified Busulfan and cyclophosphamide(BuCy) conditioning regimen including cytosine arabinoside 2 g/m2/12 h (on days −10 and −9), busulfan 0.8 mg/kg/6 h (on days −8 to −6), cyclophosphamide 1.8 g/m2/day (on days −5 and −4), and methyl N-(2-chloroethyl)-N-cyclohexyl-N-nitrosourea 250 mg/m2 (on day −3) while a very small portion of patients received fractioned TBI (200cGy Bid for 5-6 doses). Peripheral blood stem cells and/or bone marrow stem cells were collected from donors after stem cell mobilization was induced by granulocyte colony-stimulating factor.

GVHD prophylaxis for those accepting stem cells from an HLA-haploidentical or matched unrelated donor was composed of cyclosporine, mycophenolate mofetil, methotrexate, and rabbit antithymocyte globulin. For those accepting stem cells from HLA-matched related donors, only cyclosporine was administered.

Definitions and evaluations

Complete remission was defined in accordance with the National Comprehensive Cancer Network (NCCN) guidelines. Minimal residual disease (MRD) positivity was defined as absence of leukemia cells in BM as determined by flow cytometry (FCM) with a sensitivity threshold of 10-4. The definition of neutrophil recovery was an absolute neutrophil count (ANC) >0.5×109/L for 3 consecutive days. Platelet (PLT) recovery was defined as a count of PLT >20 × 109/L for 7 consecutive days without platelet infusion. Bloodstream infections (BSIs) were defined as the isolation of bacteria from at least one blood culture. The diagnosis and grading of acute and chronic GVHD were based on modified Glucksberg criteria and National Institutes of Health consensus criteria. Thrombotic microangiopathy (TMA) was based upon Jodele criteria.

Supplementary Table 1. Patient characteristics among 3 groups.

| **Characteristics** | **Single-targeting CAR T-cell**  **(n=43)** | **Dual-target CAR T-cell**  **(n=29)** | **Non-CART**  **(n=97)** | P value |
| --- | --- | --- | --- | --- |
| Gender, n(%) |  |  |  | 0.668 |
| Female | 22 (51.2%) | 12 (41.4%) | 43 (44.3%) |  |
| Male | 21 (48.8%) | 17 (58.6%) | 54 (55.7%) |  |
| Age | 28.0 [21.0;40.5] | 30.0 [21.0;38.0] | 32.0 [24.0;44.0] | 0.286 |
| Disease status pre-transplant, n(%) |  |  |  | **<0.001** |
| CR=1 | 11 (25.6%) | 6 (20.7%) | 81 (83.5%) |  |
| CR≥2 | 32 (74.4%) | 23 (79.3%) | 16 (16.5%) |  |
| Donor source, n(%) |  |  |  | 0.439 |
| Haploidentical | 32 (74.4%) | 26 (89.7%) | 72 (74.2%) |  |
| MUD | 7 (16.3%) | 1 (3.4%) | 13 (13.4%) |  |
| MSD | 4 (9.3%) | 2 (6.9%) | 12 (12.4%) |  |
| BCR-ABL fusion gene, n(%) | 15 (34.9%) | 10 (34.5%) | 38 (39.2%) | 0.839 |
| Philadelphia chromosome–like, n(%) | 6 (14.0%) | 2 (6.9%) | 1 (1.1%) | **0.004** |
| MLL-AF4, n(%) | 1 (2.3%) | 1 (3.5%) | 4 (4.1%) | 1.000 |
| Complex karyotype, n(%) | 2 (4.7%) | 0 (0.0%) | 3 (3.1%) | 0.692 |
| Hypodipoidy, n(%） | 2 (4.7%) | 2 (6.9%) | 6 (6.2%) | 1.000 |
| Donor-recipient gender match, n(%) |  |  |  | 0.109 |
| Female to male | 38 (88.4%) | 22 (75.9%) | 88 (90.7%) |  |
| Others | 5 (11.6%) | 7 (24.1%) | 9 (9.28%) |  |
| Conditioning regimen, n(%) |  |  |  | 0.92 |
| Bu-based | 39 (90.7%) | 27 (93.1%) | 90 (92.8%) |  |
| TBI-based | 4 (9.3%) | 2 (6.9%) | 7 (7.2%) |  |
| Median MNC count,×10^8^/kg | 9.84 [7.22;13.1] | 12.2 [9.41;14.8] | 11.1 [8.30;15.3] | 0.183 |
| Median CD34 count, ×10^6^/kg | 3.87 [2.97;4.94] | 4.18 [2.97;6.13] | 4.13 [3.18;5.94] | 0.258 |
| Graft type, n(%) |  |  |  | 0.292 |
| BM | 2 (4.6%) | 0 (0.0%) | 2 (2.0%) |  |
| BM+PB | 18 (41.9%) | 8 (27.6%) | 28 (28.9%) |  |
| PB | 23 (53.5%) | 21 (72.4%) | 67 (69.1%) |  |
| ABO compatibility, n(%) |  |  |  | 0.280 |
| Bidirectional mismatch | 4 (9.30%) | 3 (10.3%) | 6 (6.19%) |  |
| Major mismatch | 3 (7.0%) | 6 (20.7%) | 20 (20.6%) |  |
| Minor mismatch | 7 (16.3%) | 4 (13.8%) | 22 (22.7%) |  |
| Match | 29 (67.4%) | 16 (55.2%) | 49 (50.5%) |  |
| MRD before HSCT, n(%) |  |  |  | 0.804 |
| Negative | 37 (86.0%) | 24 (82.8%) | 74 (76.3%) |  |
| Positive | 4 (9.30%) | 3 (10.3%) | 15 (15.5%) |  |
| Missing data | 2 (4.65%) | 2 (6.90%) | 8 (8.25%) |  |
| Time to transplantation after CAR-T, days, Median | 62.0 [47.5;79.5] | 60.0 [55.0;79.0] |  | 0.638 |

Supplementary Table 2. Transplant outcomes.

| **Parameter** | **Non-CAR-T group** | **Single targeting CAR-T group** | **Dual targeting CAR-T group** |
| --- | --- | --- | --- |
| **Median days of neutrophil engraftment(range)** | 12(8-37) | 12(10-20) | 15(10-19) |
| **Median days of platelet engraftment(range)** | 14(10-109) | 11(11-116) | 17(10-85) |
| **Bloodstream infection at day 30, %(95% CI)** | 7.2(1.9, 12.2) | 14.0(2.9, 23.7) | 17.2(2.3, 29.9) |
| **CMV viremia at day 100, %(95% CI)** | 28.6(18.9, 37.2) | 39.5(23.0, 52.5) | 65.5(43.1, 79.1) |
| **EBV viremia at day 100, %(95% CI)** | 2.1(0, 5.0) | 18.9(6.2, 29.8) | 10.3(0, 20.8) |
| **Acute GVHD at day 100,%(95% CI)** | 24.4(15.2, 32.6) | 16.5(4.5, 26.9) | 31.0(12.0, 46.0) |
| **Chronic GVHD at day 360, %(95% CI)** | 18.3(9.7, 26) | 11.2(0.2, 21.1) | 20.0(2.6, 34.3) |
| **1-year incidence of relpase, %(95% CI)** | 12.2(4.8, 19.0) | 16.5(3.4, 27.9) | 25.0(7.0, 39.6) |
| **1-year incidence of NRM, %(95% CI)** | 9.6(3.4, 15.3) | 4.6(0, 11.3) | 3.4(0, 10.2) |
| **1-year probability of OS, %(95% CI)** | 90.4(84.7, 96.6) | 90.3(81.8, 99.8) | 92.7(83.5, 100) |
| **1-year probability of LFS, %(95% CI)** | 82.8(75.4, 90.8) | 79.3(67.3, 93.3) | 72.1(57.3, 90.6) |

**SUPPLEMENTARY NOTES**

Incidence of GVHD, Bloodstream infection and TMA

The cumulative incidence of grade II-IV acute GVHD was similar between the 3 groups with no statistical significance (**Fig. 1e**; p=0.33). There was also no significant difference in extensive chronic GVHD between the 3 groups (**Fig. 1f**; p=0.54).

The cumulative incidence of BSI infection by day 30 after HSCT was not significantly different between the 3 groups (**Fig. 1g**; p=0.21). The cumulative incidence of TMA by day 100 after transplantation was also similar across the 3 groups (**Fig. 1h**; p=0.56).

Prognosis

The cumulative incidence of relapse in patients between the 3 groups did not differ significantly (**Fig. 1i**; p=0.16). Cumulative incidence of non-relapse mortality was not different among the 3 groups (NRM; **Fig 1j**; p=0.45). LFS and OS at 1 year were also similar across the 3 groups (p=0.40 and p=0.91, respectively; **Fig 1k; Fig 1l**).

Significance

Although CAR-T therapy has emerged as a promising therapy, capable of achieving CR in R/R ALL, remissions can be short-lived in a large portion of patients (12). Sequential allo-HSCT may improve the durability of remission for this subset of patients (13). Data are available for the safety and favorable efficacy of this novel combination therapy, while little attention has been paid to the impact this combinational therapy would have on transplantation-related complications (14).

As prolonged thrombocytopenia has been associated with life-threatening complications, a higher TRM, and an lower OS, our results indicated that some candidate strategies may be used for stimulating megakaryopoiesis after integration of CAR-T therapy and allo-HSCT(25). Based on our data, patients receiving CAR-T therapy bridging to HSCT should be regarded as high risk for CMV and EBV infection, multiple factors may act synergistically, disturbing immune reconstitution, leading to high risk of CMV- and EBV-viremia within the combinational therapy which bears further attention in biologic and clinical research. Prophylaxis should be considered. It has been suggested that monthly assessment of lymphocyte subsets (CD4+) and immunoglobulins (IGs) should be monitored until the 6th month after CAR-T cell infusion alone for hypogammaglobulinemia (35). It is also recommended that IgG replacement be considered in patients receiving CAR-T cell infusion with severe hypogammaglobulinemia to prevent further infection (36, 37). Thus, it may be necessary to monitor the lymphocyte subsets and IGs after CAR-T therapy bridging to HSCT as well as providing patients with IGs if necessary to lower the risk of infection.

Limitations

There are limitations to this study. The retrospective nature has inherent risks and bias. As CAR-T therapy has achieved compelling success in R/R B-ALL, we were unable to secure enough patients with R/R B-ALL without CAR-T therapy for the non-CAR-T group. There were more patients with R/R B-ALL and high-risk cytogenetics in the CAR-T group. Nevertheless, the unbalanced baseline characteristic with pretransplant disease status was corrected and adjusted to avoid bias through univariate and multivariate statistical analysis. Other baseline characteristics that may have influence on transplant related complications in the CAR-T and non-CAR-T groups were comparable with no statistical significance. In addition, the choice of covariates for the multivariate analysis was limited by the small number of observed events.
